# Clinical efficacy and cost-effectiveness of endobronchial ultrasound-guided transbronchial needle aspiration for preoperative staging of non-small–cell lung cancer: Results of a French prospective multicenter trial (EVIEPEB)

**DOI:** 10.1371/journal.pone.0208992

**Published:** 2019-01-07

**Authors:** Christos Chouaid, Mathieu Salaün, Valérie Gounant, Michel Febvre, Jean-Michel Vergnon, Vincent Jouniaux, Clément Fournier, Samy Lachkar, Christophe Hermant, Christophe Raspaud, Xavier Quantin, Jean-Jacques Quiot, Anita Molard, Charles Dayen, Charles-Hugo Marquette, Hervé Lena, Gérard Zalcman, Luc Thiberville

**Affiliations:** 1 Department of Pneumology, CHI Créteil, Créteil, France; 2 Department of Pneumology, CHU de Rouen, Rouen, France; 3 Department of Pneumology CHU Bichat, APHP, Paris, France; 4 Department of Pneumology, CHU Tenon, APHP, Paris, France; 5 Department of Pneumology, CHU Saint-Etienne, Saint-Etienne, France; 6 Department of Pneumology, CHU d’Amiens, Amiens, France; 7 Department of Pneumology CHU de Lille, Lille, France; 8 Department of Pneumology, CHU de Toulouse, Toulouse, France; 9 Clinique Pasteur Toulouse, Toulouse, France; 10 Department of Pneumology, CHU Montpellier, Montpellier, France; 11 Department of Pneumology, CHU Brest, Brest, France; 12 Department of Pneumology, CHU Strasbourg, Strasbourg, France; 13 Department of Pneumology, CH Saint-Quentin, Saint-Quentin, France; 14 Department of Pneumology, CHU Nice, Nice, France; 15 Department of Pneumology, CHU de Rennes, Rennes, France; University of Michigan, UNITED STATES

## Abstract

This two-step study evaluated the cost-effectiveness of endobronchial ultrasound-guided transbronchial needle aspiration (EBUS-TBNA) for presurgery staging of non-small cell lung cancer (NSCLC) in France (EVIEPEB; ClinicalTrial.gov identifier NCT00960271).

Step 1 consisted of a high-benchmark EBUS-TBNA–training program in participating hospital centers. Step 2 was a prospective, national, multicenter study on patients with confirmed or suspected NSCLC and an indication for mediastinal staging with at least one lymph node > 1 cm in diameter. Patients with negative or uninformative EBUS-TBNA and positron-emission tomography-positive or -negative nodes, respectively, underwent either mediastinoscopy or surgery. Direct costs related to final diagnosis of node status were prospectively recorded.

Sixteen of 22 participating centers were certified by the EBUS-TBNA–training program and enrolled 163 patients in Step 2. EBUS-TBNA was informative for 149 (91%) patients (75 malignant, 74 non-malignant) and uninformative for 14 (9%). Mediastinoscopy was avoided for 80% of the patients. With a 52% malignant-node rate, EBUS-TBNA positive- and negative-predictive values, respectively, were 100% and 90%. EBUS-TBNA was cost-effective, with expected savings of €1,450 per patient, and would have remained cost-effective even if all EBUS-TBNAs had been performed under general anesthesia or the cost of the procedure had been 30% higher (expected cost-saving of €994 and €1,427 per patient, respectively).

After EBUS-TBNA training and certification of participating centers, the results of this prospective multicenter study confirmed EBUS-TBNA cost-effectiveness for NSCLC staging.

## Introduction

Mediastinoscopy has traditionally been considered the gold standard for biopsying lymph nodes of patients with suspected or confirmed lung cancer. However, mediastinoscopy requires general anesthesia, provides access to only the paratracheal and anterior subcarinal lymph nodes, and often requires hospitalization. Rare but potentially severe complications of mediastinoscopy include cord palsy, innominate vein damage and even death [1].

The advent of endobronchial ultrasound-guided transbronchial needle aspiration (EBUS-TBNA), during the early 1990s in Japan, radically changed the guidelines for lung-cancer mediastinal node staging. Today, EBUS-TBNA has been shown to facilitate biopsy of Mountain and Dressler classification [2] lymph-node stations 2 (upper paratracheal), 4 (lower paratracheal), 7 (subcarinal), 10 (hilar) and 11 (interlobar) of the, usually under light sedation and as a day-hospital procedure [3–7].

Few “real-world” studies have explicitly evaluated the role and cost implications of EBUS-TBNA in patients with mediastinal lymphadenopathy and suspected or confirmed malignancy [8–12]. In 2007, few French centers were able to offer this non-invasive procedure. Therefore, the French Pulmonology Society (SPLF) and French National Cancer Institute (Inca) launched a nationwide multicenter two-step preoperative EBUS-TBNA trial. The objective of Step 1 was to enroll specialized centers in a high-benchmark EBUS-TBNA–training program. Step 2 was designed to assess EBUS-TBNA cost-effectiveness in a real-world setting.

## Methods

### Study design

#### Step 1: Training program

In study Step 1, all participating centers received an EBUS system (BF-UC160F-OL8; Olympus, Keymed, UK) and dedicated puncture kits sufficient for 40 procedures free-of-charge, provided that they accepted prospective evaluation of all subsequent procedures. All the investigators underwent 2 days of high-benchmark training to learn the EBUS-TBNA procedure and were certified for study Step 2 if, during a 6-month period, at least nine of their 10 consecutive procedures were informative (tissue biopsies containing lymphocytes or tumor cells in at least one station). Several investigators could be certified in each center.

All consecutive eligible patients were informed of the protocol, gave their signed informed consent to participate and were enrolled in a central database before the procedure. The main inclusion criteria were suspected or confirmed lung cancer, suspicious mediastinal lymph node(s), and the use of EBUS-TBNA as the initial diagnostic procedure for node assessment.

The procedure was done according to local practices: some centers used brief general anesthesia with day hospitalization, others did it on an outpatient basis with intravenous sedation (midazolam and fentanyl in addition to topical lidocaine) and some centers mixed the two practices. The system’s linear echoendoscope was always used, and all hilar and mediastinal lymph nodes were systematically assessed. Under ultrasound guidance, mediastinal and/or hilar lymph nodes were punctured with a 22-gauge needle and suction was applied. Tissue samples were transferred to glass slides and air-dried or directly expelled into a liquid fixative for cell-block processing. Any tissue cores were placed in formalin. The lymph-node stations and numbers sampled, and the number of passes per node were left to the investigator’s discretion. Biopsies were evaluated on-site in some centers. The pathologists were provided with clinical information, reflecting routine clinical practice. An informative biopsy was defined as containing lymphoid tissue or tumor cells.

#### Step 2: EBUS-TBNA cost effectiveness

Centers with a least one certified investigator were allowed to participate in Step 2, which consisted of a multicenter single-arm prospective trial designed to assess EBUS-TBNA cost-effectiveness in a real-world setting. The inclusion criteria for this step were suspected or confirmed non-small–cell lung cancer (NSCLC) with suspected mediastinal lymph-node involvement, eligibility for surgery with curative intent, adequate fitness for EBUS-TBNA and surgery, and no evidence of metastatic disease. The exclusion criteria were previous lung-cancer treatment, concurrent malignancy, uncorrected coagulopathy and unsuitability for surgical staging. In each center, between March 2009 and June 2011, consecutive patients with mediastinal lymphadenopathy and suspected or confirmed lung cancer were invited to participate in the trial. All recruited patients underwent EBUS-TBNA. Tolerance was assessed using a visual analogue scale. EBUS-TBNA complications were recorded. In each center, patient-management decisions following the EBUS-TBNA results were decided by a multidisciplinary team that included radiologists, pulmonologists, oncologists, thoracic surgeons and pathologists. If EBUS-TBNA did not yield a diagnosis retained by the multidisciplinary team, the patient underwent cervical mediastinoscopy under general anesthesia via an incision above the suprasternal notch, with sampling of lymph-node stations 2, 4 and 7.

Step-2 primary endpoints were the percentages of patients spared mediastinoscopy and EBUS-TBNA impact on healthcare costs, compared to systematic mediastinoscopy as the first diagnostic procedure.

### Economic analyses

The economic analysis, limited to direct costs, adopted the perspective of the French healthcare system. The cost of the EBUS-TBNA strategy (EBUS-TBNA for all patients, followed by mediastinoscopy if no lymph-node diagnosis was obtained, was compared to that of a front-line mediastinoscopy strategy. We assumed that there would be no mediastinoscopy complications, and post-diagnostic treatments and their outcomes would be the same, regardless of the diagnostic strategy.

Healthcare-resource consumption was assessed prospectively from the case-report forms. All the resources used were collected and added together. Costs were derived from national tariffs for diagnosis-related groups and national fees for ambulatory care, as provided by the French Ministry of Health and the French National Health Insurance program [13, 14]. The costs of EBUS-TBNA with local anesthesia, EBUS-TBNA with general anesthesia and mediastinoscopy were, respectively, €120, €840 and €2,300. Sensitivity analyses to test the strength of the economic analysis were computed by varying the frequencies of metastatic node disease and EBUS-TBNA procedures performed under general anesthesia, and by raising the EBUS-TBNA cost by 30%. This 30% threshold was chosen because the cost of the procedure in France is <30% of its mean cost in other European countries. We also calculated the EBUS-TBNA threshold for cost-equivalence of the two strategies.

### Statistical analyses

Demographic and clinical characteristics of the study population are summarized as means ± standard deviation, medians or numbers (%), depending on their type and distribution. The accuracies of EBUS-TBNA and mediastinoscopy were calculated, in terms of their sensitivity and negative-predictive value, for the entire population and the subgroups with positive- or negative positron-emission tomography (PET) scans. The design, running, analysis and reporting of this study conformed to the Standard of Reporting Diagnostic Accuracy Guidelines [15]. The Nord-Ouest Research Ethics Committee, France, approved the study protocol on 26 March 2010. The ClinicalTrial.gov identifier is NCT00960271.

## Results

Twenty-two centers (six already equipped) volunteered for study Step 1 and were included in the training program. Three centers did not include patients during the first 6 months and their participation was stopped. The other 19 centers included 231 patients (200 by principal investigators and 31 by sub-investigators). Most patients were men (77%), mean age was 61±11.4 (range 37–86) years, and 78% of the patients were smokers or ex-smokers. At inclusion, 100% and 53% of the patients, respectively, had had computed-tomography (CT) and PET scans. The mean number of CT-detected pathological lymph nodes was 2.2 per patient. EBUS-TBNA was performed under general anesthesia, conscious sedation or local single midazolam injection, respectively, in 75%, 9% and 16% of the patients. The EBUS-TBNA procedure took a median of 47 minutes. Patient-rated tolerability was rated good, medium or poor, respectively, for 90%, 10% and 0.4% of them. A pathologist was present during the procedure in five centers. Seventy-six percent (387/508) of lymph nodes identified as pathological by imaging were biopsied, with a median of three punctures per node; a median of 2 (range 1–4) nodes were punctured per procedure, and the mean size of the sampled nodes was 1.5±0.4 cm. Stations 7 and 4R were biopsied significantly more often (Table 1). Sampling was independent of node size. A total of 83% of the punctures were informative, which was significantly associated with the number of punctures performed (Table 2) and lymph-node size, but not lymph-node station (Table 2). At least one informative puncture was obtained from 206/231 (89%) patients (Table 3).

**Table 1:** Mediastinal lymph-node puncture rates according to station (study Step 1).

Station	Not sampled (*n* = 121)	Sampled nodes (*n* = 387)	Total (*n* = 508)	*P*
2R	11 (38%)	18 (62%)	29	<0.001*
4R	33 (24%)	102 (76%)	135	
10R	16 (38%)	26 (62%)	42	
11R	16 (27%)	44 (73%)	60	
7	10 (7%)	137 (93%)	147	
2L	5 (100%)	0 (0%)	5	
4L	15 (33%)	30 (67%)	45	
10L	8 (53%)	7 (47%)	15	
11L	7 (23%)	23 (77%)	30	

All locations combined

**Table 2:** EBUS-TBNA node-biopsy results according to station, the number of punctures and node size (study Step 1).

Parameter	Uninformative (*n* = 69)	Informative (*n* = 318)	Total (*n* = 387)	*P*
Station, *n* (%)				
2R	1 (6%)	17 (94%)	18	0.24
4R	22 (22%)	80 (78%)	102	
10R	3 (12%)	23 (88%)	26	
11R	9 (20%)	35 (80%)	44	
7	20 (15%)	117 (85%)	137	
4L	8 (27%)	22 (73%)	30	
10L	3 (43%)	4 (57%)	7	
11L	3 (13%)	20 (87%)	23	
Punctures per node, *n* (%)				
<3	35 (26%)	100 (74%)	135	0.002
3	34 (13%)	218 (87)	252	
CT-determined node diameter (cm)				
Mean±SD	1.4±0.4	1.6±0.6		0.02
Median [range]	1.4 [0.6–3]	1.5 [0.5–7.5]		

CT: thoracic computed tomography

**Table 3:** **EBUS-TBNA diagnostic yield in study Step 1:** Based on 231 patients.

Diagnostic yield	*n* (%)
Informative	206 (89)
Squamous cell lung carcinoma	25 (12)
Lung adenocarcinoma	35 (17)
Large-cell lung carcinoma	21 (10)
Small-cell lung cancer	12 (6)
Lymphoma	2 (1)
Other malignancies	6 (3)
Non-malignant^a^	11 (5)
Normal	94 (46)
Uninformative	25 (11%)

^a^Eight sarcoidosis, 2 tuberculosis and 1 silicosis.

At the end of Step 1, three centers had not achieved the target of nine informative out of 10 consecutive procedures. These three centers had no previous experience with EBUS-TBNA. A central cytology review confirmed that their failures reflected the TBNA technique and not cytological processing. The operators’ previous experience was significantly associated with the time taken to reach the 9/10 target. The 13 investigators who reached the target had performed 33.3±31 procedures before the training program, compared to 5.3±4.3 procedures for those who failed to reach the target. After Step 1, 16 centers and 20 investigators were approved to participate in Step 2.

During Step 2, the 16 participating centers included 163 patients with suspected or confirmed lung cancer and a theoretical indication for staging mediastinoscopy (Table 4). Almost all the patients had a PET scan at enrollment in Step 2. During this step, 75% of the procedures were performed without general anesthesia. Also, a pathologist was more frequently present during Step 2 procedures (18% vs 10% in Step 1). The procedure was well-tolerated, with only four complications, including three reversible laryngospasms. Those complications did not lead to early termination of the procedure and not post procedure admission was observed.

**Table 4:** **Study Step 2:** Characteristics of 163 patients.

Characteristic	Value
Male sex	133 (82%)
Age (years)	63.2±11.6
Smoking status	
Smoker	86 (53%)
Former smoker	68 (42%)
Never smoker	9 (6%)
Tests before EBUS-TBNA	
Thoracic CT scan	163 (100%)
Abdominal CT scan	122 (75%)
Brain CT scan	119 (73%)
Pulmonary function tests	144 (88%)
Flexible fibroscopy	159 (98%)
FDG-PET scan	162 (99%)
Anesthesia	
Local	27 (17%)
Midazolam sedation	44 (27%)
General	90 (55%)
Length of the procedure (hours)	1.2±0.6
On-site pathologist	30 (18%)
Tolerability	
No adverse event	159 (98%)
Grade 1 chest pain	1 (0.6%)
Grade 1 laryngospasm	3 (2%)

CT, computed tomography; PET, positron-emission tomography.

Results are expressed as *n* (%) or mean±SD.

**Fig 1:**
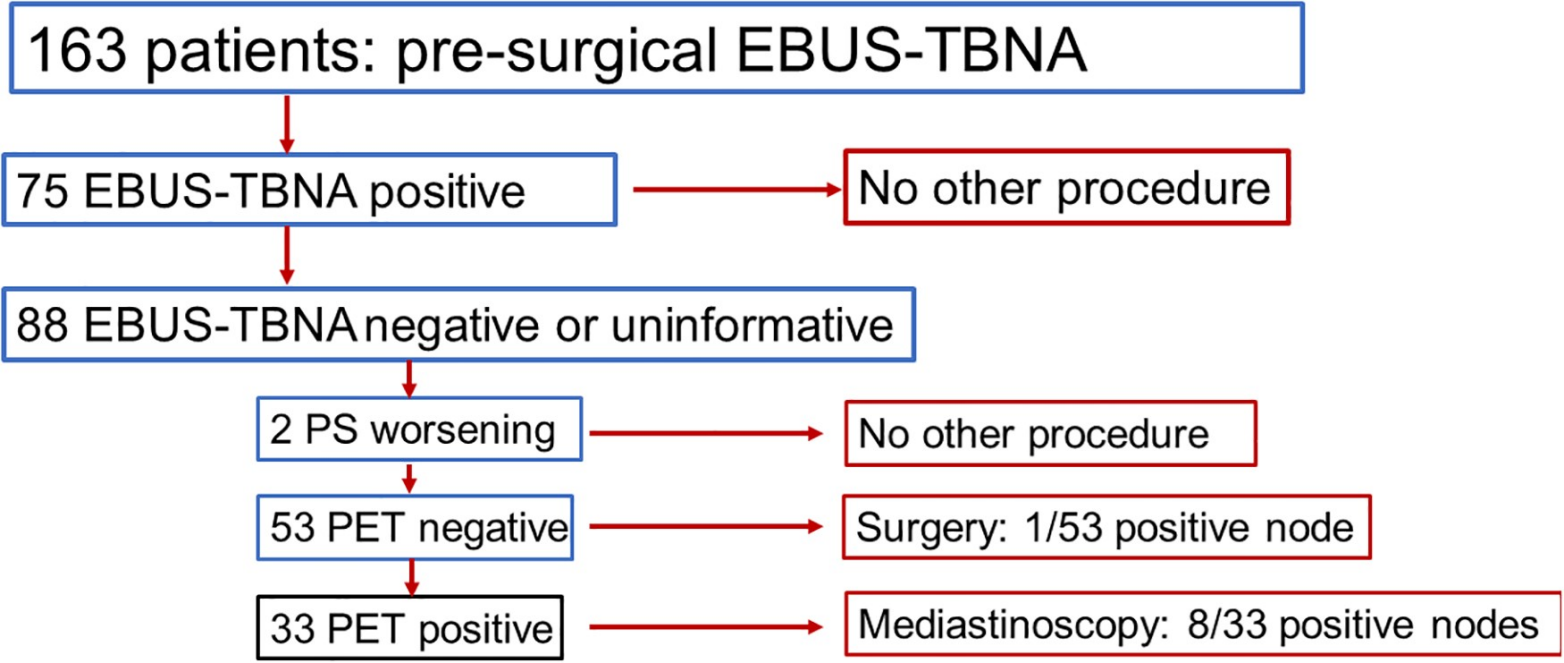
**Flow chart of the study (EBUS-TBNA:** endobronchial ultrasound-guided transbronchial needle aspiration, EBUS-TBNA positive: tumors cells, EBUS-TBNA negative: Lymphocytes and no tumor cells, PS: performans status, PET positive: significant uptake, PET negative: no significant uptake,.

EBUS-TBNA histological examinations found tumor cells in 75 patients, normal node cells in 74 and were uninformative for 14 (Table 5). Among the 88 patients with normal node cells or uninformative results, two (one indeterminate and one normal) experienced rapid tumor progression during the days following the procedure and could not be included in the accuracy analysis. Fifty-three EBUS-TBNA–and mediastinal PET-scan–negative patients underwent surgery, confirming the absence of nodal tumor cells in all but one (Fig 1). Thirty-three EBUS-TBNA–negative and PET-scan–positive patients underwent mediastinoscopy, which yielded a final diagnosis of malignancy in eight. EBUS-TBNA avoided mediastinoscopy for 128/161 (80%) patients. EBUS-TBNA had 89.3% sensitivity and 100% specificity. With a 52% malignant-node rate, the positive- and negative-predictive values of this strategy were 100% and 89.5%, respectively. EBUS-TBNA–identified malignant-node frequencies, sensitivities and negative-predictive values, respectively, were 77%, 90.4% and 75.7%, respectively, for 105 PET-scan–positive patients and 2%, 83.3% and 96.6% for 58 PET-scan–negative patients.

**Table 5:** Costs of the mediastinoscopy and EBUS-TBNA^a^ strategies.

Procedure	Mediastinoscopy *n* = 163	EBUS-TBNA *n* = 163
EBUS (local anesthesia)		103 × €120
EBUS (general anesthesia)		60 × €840
Mediastinoscopy	163 × €2300	33 × €2300
Total cost (cost/patient)	€374,900 (€2300)	€138,660 (€850)

EBUS-TBNA, endobronchial ultrasound-guided transbronchial node aspiration.

^a^EBUS-TBNA was followed by mediastinoscopy only when uninformative or normal for a PET-scan–positive patient.

The respective total costs of the mediastinoscopy and EBUS-TBNA strategies were €370,330 and €138,600 (Table 5). EBUS-TBNA was cost-effective, with expected savings of €1,450 per patient. That cost-saving depended on the disease rate, ranging from €1,294 per patient for 30% to €1,703 for 80%. The EBUS-TBNA strategy would have remained largely cost-effective, even if all the procedures had been performed under general anesthesia (expected savings €994 per patient) and the EBUS-TBNA cost had been 30% higher (expected savings €1,427 per patient). Cost-equivalence of the two strategies was obtained for an EBUS-TBNA cost of €1834.

## Discussion

This multicenter study led to EBUS-TBNA equipment to being installed in 22 hospitals and enabled us to evaluate the procedure in the real world setting. A medical-economic evaluation was also conducted in the setting of the French healthcare system.

One original feature of this study was the use of a high-benchmark training program with a simple and explicit target, namely nine informative out of 10 consecutive procedures, leading to the certification of 20 operators. The operators’ learning curves were significantly associated with their prior experience with EBUS-TBNA, as observed previously [5, 6, 16–18]. Those experts consider that trainees need to perform 50 EBUS procedures to become competent. However, that expert opinion was based on only a few studies that addressed this issue indirectly. For example, analysis of the first 215 EBUS-TBNA procedures performed by two physicians who were already experienced bronchoscopists showed that cumulative sensitivity started to plateau after 50 procedures but continued to improve between 50 and 100 procedures [6]. In agreement with these results, in our study, the 13 investigators who reached the target had performed 33.3±3 procedures before the training program, compared to 5.3±4 procedures for those who failed to reach the target. In agreement with a previous report [19], we found that the number of nodes punctured per patient was an important predictor of success based on “informative” results of the procedure. In that study on 286 patients, informative results were obtained for 95.3% of patients when at least two stations were punctured, compare to 84.4% when only one nodal station was biopsied.

The average number of 2.8 punctures per node herein was in keeping with current recommendations [20]; it is well-established that a higher number of punctures enhances the success of the procedure. That tenet was clearly demonstrated in a study on NSCLC patients, whose success rates were 90.1% for one and 100% for three punctures. Lymph-node size also significantly influenced the success of the procedure [20].

One advantage of this study is its pragmatic approach, in near real-life conditions. The centers were free to adopt their regular practices regarding the type of anesthesia, the presence of an on-site pathologist and an outpatient setting. No association was found between any of these variables and the success of the procedure.

The clinical value of the EBUS-TBNA strategy was assessed during study Step 2. We found that EBUS-TBNA avoided mediastinoscopy for 80% of the patients, a rate similar to the 87% obtained in a prospective study conducted in the UK, in the same context [21]. Similarly, EBUS-TBNA sensitivity and specificity in our study are in keeping with values reported elsewhere [21–25].

The cost-minimization approach was considered most appropriate for this study, as the same final diagnosis, treatment and outcomes would have been reached, regardless of whether the diagnosis was based on initial EBUS-TBNA or mediastinoscopy. We found that EBUS-TBNA was cost-effective, with expected savings of €1,450 per patient, and that it would have remained largely dominant even if all the procedures had been performed under general anesthesia. These results are in keeping with published economic analyses [12, 26]. One of the main drivers of this result is the EBUS-TBNA tariff. In France, this procedure costs €120, which seems to be undervalued by comparison with the UK [25], Singapore [9], the USA [1], and Australia [25]. In terms of cost-accounting, the hospital charge for an EBUS-TBNA procedure was €250 euros, excluding personnel costs, equipment depreciation, maintenance and repair. A Canadian study highlighted the importance of maintenance and repair costs with a valuation of CAN$116 per EBUS-TBNA procedure, compared to CAN$21 for flexible bronchoscopy [27]. Undervaluation of the EBUS-TBNA tariff remains one of the main obstacles to widespread implementation of this procedure in French hospitals.

This study took place in high-volume tertiary centers following a high-benchmark training program in which EBUS-TBNA was performed by experienced physicians assisted by experienced pathologists. As a result, the EBUS-TBNA sensitivity obtained herein and the number of mediastinoscopies avoided might not be immediately reproducible in other centers.

In summary, EBUS-TBNA is a safe, highly sensitive and cost-saving procedure for the diagnosis and staging of NSCLCs. In France, EBUS-TBNA is now firmly established in NSCLC-management algorithms.

## References

[1] Porte H, Roumilhac D, Eraldi L, Cordonnier C, Puech P, Wurtz A. The role of mediastinoscopy in the diagnosis of mediastinal lymphadenopathy. Eur J Cardiothorac Surg. 1998;13:196–199. 9583827 10.1016/s1010-7940(97)00324-2

[2] Mountain CF, Dressler CM. Regional lymph node classification for lung cancer staging. Chest. 1997;111:1718–1723. 9187199 10.1378/chest.111.6.1718

[3] Colt HG, Davoudi M, Murgu SD. Scientific evidence and principles for the use of endobronchial ultrasound and transbronchial needle aspiration. Expert Rev Med Devices. 2011;8:493–513. 10.1586/erd.11.14 21728734

[4] Currie GP, McKean ME, Kerr KM, Denison AR, Chetty M. Endobronchial ultrasound-transbronchial needle aspiration and its practical application. QJM. 2011;104:653–662. 10.1093/qjmed/hcr071 21546452

[5] Ernst A, Silvestri GA, Johnstone D. American College of Chest Physicians. Interventional pulmonary procedures: guidelines from the American College of Chest Physicians. Chest. 2003;123:1693–1717. 12740291 10.1378/chest.123.5.1693

[6] Miller D, Mahendra P, Bruce V, Kerr KM, McKean M, Chetty M, et al Endobronchial ultrasound transbronchial needle aspiration at Aberdeen Royal Infirmary: the initial experience. QJM. 2012;105:607–608. 10.1093/qjmed/hcs049 22396604

[7] Ye T, Hu H, Luo X, Chen H. The role of endobronchial ultrasound guided transbronchial needle aspiration (EBUS-TBNA) for qualitative diagnosis of mediastinal and hilar lymphadenopathy: a prospective analysis. BMC Cancer 2011;11:100 10.1186/1471-2407-11-100 21418631 PMC3076261

[8] Andrade RS, Podgaetz E, Rueth NM, Majumder K, Hall E, Saric C, et al Endobronchial ultrasonography versus mediastinoscopy: a single-institution cost analysis and waste comparison. Ann Thorac Surg. 2014;98:1003–1007. 10.1016/j.athoracsur.2014.04.104 25038020

[9] Ang SY, Tan RW, Koh MS, Lim J. Economic analysis of endobronchial ultrasound (EBUS) as a tool in the diagnosis and staging of lung cancer in Singapore. Int J Technol Assess Health Care. 2010;26:170–174. 10.1017/S0266462310000176 20392320

[10] Callister ME, Gill A, Allott W, Plant PK. Endobronchial ultrasound guided transbronchial needle aspiration of mediastinal lymph nodes for lung cancer staging: a projected cost analysis. Thorax. 2008; 63:384. 10.1136/thx.2007.090308 18364451

[11] Harewood GC, Pascual J, Raimondo M, Woodward T, Johnson M, McComb B, et al Economic analysis of combined endoscopic and endobronchial ultrasound in the evaluation of patients with suspected non-small cell lung cancer. Lung Cancer. 2010;67:366–371. 10.1016/j.lungcan.2009.04.019 19473723 PMC2822087

[12] Sharples LD, Jackson C, Wheaton E, Griffith G, Annema JT, Dooms C, et al Clinical effectiveness and cost-effectiveness of endobronchial and endoscopic ultrasound relative to surgical staging in potentially resectable lung cancer: results from the ASTER randomised controlled trial. Health Technol Assess. 2012;16:1–75. 10.3310/hta16180 22472180

[13] Ministère de la Santé et des Solidarités. Arrêté du 27 février 2007 relatif à la classification et à la prise en charge des prestations d’hospitalisation pour les activités de médecine, chirurgie, obstétrique et odontologie et pris en application de l’article L 162-22-6 du code de la sécurité sociale. Journal Officiel n°50 du 28 février 2007, texte 72.

[14] Nomenclature Générale des Actes Professionnels. Union des Caisses Nationales de Sécurité Sociale (UCANSS). Paris. http://www.ameli.fr/fileadmin/user_upload/documents/NGAP-oct-2009.pdf

[15] Bossuyt PM, Reitsma JB, Bruns DE, Gatsonis CA, Glasziou PP, Irwig LM, et al Towards complete and accurate reporting of studies of diagnostic accuracy: the STARD initiative. BMJ. 2003;326:41–44. 12511463 10.1136/bmj.326.7379.41 PMC1124931

[16] Davoudi M, Colt HG, Osann KE, Lamb CR, Mullon JJ. Endobronchial ultrasound skills and tasks assessment tool: assessing the validity evidence for a test of endobronchial ultrasound-guided transbronchial needle aspiration operator skill. Am J Respir Crit Care Med. 2012;186:773–779. 10.1164/rccm.201111-1968OC 22837376

[17] Wahidi MM, Silvestri GA, Coakley RD, Ferguson JS, Shepherd RW, Moses L, et al A prospective multi-center study of competency metrics and educational interventions in the learning of bronchoscopy among starting pulmonary fellows. Chest. 2010;137:1040–1049. 10.1378/chest.09-1234 19858234

[18] Unroe MA, Shofer SL, Wahidi MM. Training for endobronchial ultrasound: methods for proper training in new bronchoscopic techniques. Curr Opin Pulm Med. 2010;16:295–300. 10.1097/MCP.0b013e32833a047a 20531196

[19] McComb BL, Wallace MB, Pascual JM, Othman MO. Mediastinal staging of non small cell lung carcinoma by endoscopic and endobronchial ultra-sound–guided fine needle aspiration. J Thorac Imaging. 2011;26:147–161. 10.1097/RTI.0b013e3182171dc9 21508736

[20] Lee HS, Lee GK, Lee HS, Kim MS, Lee JM, Kim HY, et al Real-time endobronchial ultrasound-guided transbronchial needle aspiration in mediastinal staging of non-small cell lung cancer: how many aspirations per target lymph node station? Chest. 2008;134:368–374. 10.1378/chest.07-2105 18263688

[21] Navani N, Lawrence DR, Kolvekar S, Hayward M, McAsey D, Kocjan G, et al Endobronchial ultrasound–guided transbronchial needle aspiration prevents mediastinoscopies in the diagnosis of isolated mediastinal lymphadenopathy: a prospective trial. Am J Respir Crit Care Med. 2012;186:255–260. 10.1164/rccm.201203-0393OC 22652031 PMC3423452

[22] Navani N, Brown JM, Nankivell M, Woolhouse I, Harrison RN, Jeebun V, et al Suitability of endobronchial ultrasound-guided transbronchial needle aspiration specimens for subtyping and genotyping of non-small cell lung cancer: a multicenter study of 774 patients. Am J Respir Crit Care Med. 2012;185:1316–1322 10.1164/rccm.201202-0294OC 22505743 PMC3378660

[23] Um SW, Kim HK, Jung SH, Han J, Lee KJ, Park HY, et al Endobronchial ultrasound versus mediastinoscopy for mediastinal nodal staging of non-small-cell lung cancer. J Thorac Oncol. 2015;10:331–337. 10.1097/JTO.0000000000000388 25611227

[24] Hwangbo B, Kim SK, Lee HS, Lee HS, Kim MS, Lee JM, et al Application of endobronchial ultrasound-guided transbronchial needle aspiration following integrated PET/CT in mediastinal staging of potentially operable non-small cell lung cancer. Chest. 2009;135:1280–1287. 10.1378/chest.08-2019 19118267

[25] Steinfort DP, Liew D, Conron M, Hutchinson AF, Irving LB. Cost-benefit of minimally invasive staging of non-small cell lung cancer: a decision tree sensitivity analysis. J Thorac Oncol. 2010;5:1564–1570 10.1097/JTO.0b013e3181e8b2e6 20871261

[26] Medford AR, Agrawal S, Free CM, Bennett JA. A performance and theoretical cost analysis of endobronchial ultrasound-guided transbronchial needle aspiration in a UK tertiary respiratory centre. QJM. 2009;102:859–864. 10.1093/qjmed/hcp136 19789210

[27] Hergott CA, Maceachern P, Stather DR, Tremblay A. Repair costs for endo bronchial ultrasound bronchoscopes. J Bronchology Interv Pulmonol. 2010;17:223–227. 10.1097/LBR.0b013e3181e77280 23168887

